# Development of local anesthetic drug delivery system by administration of organo-silica nanoformulations under ultrasound stimuli: *in vitro* and *in vivo* investigations

**DOI:** 10.1080/10717544.2020.1856220

**Published:** 2020-12-21

**Authors:** Rong-Qin Qi, Wei Liu, Duan-Yu Wang, Fan-Qing Meng, Hong-Ying Wang, Hai-Yan Qi

**Affiliations:** a Department of Anesthesiology, Jinan Maternal and Child Health Hospital, Jinan, China; b Department of Anesthesiology, Jinan Central Hospital Affiliated to Shandong University, Jinan, China

**Keywords:** Chitosan, silica nanoparticles, anesthetic, drug delivery, biocompatibility

## Abstract

The development of local anesthetic (LA) system is the application of commercial drug for the pain management that indorses the reversible obstructive mechanism of neural transmission through preventing the innervation process in human peripheral nerves. Ropivacaine (RV) is one of the greatest frequently used LA s with the actions of long-lasting and low-toxicity for the post-operative pain management. In this work, we have approached novel design and development of glycosylated chitosan (GCS) encapsulated mesoporous silica nanoparticles (GCS-MONPs)-based nano-scaffold for sustainable distributions and controlled/supported arrival of stacked RV for targeting sites, which can be activated by either outer ultrasound activating to discharge the payload, foundation on-request and dependable analgesia. The structural and morphology analyses result established that prepared nano-formulations have successful molecular interactions and RV loaded spherical morphological structures. The drug release profile of developed nanostructure with ultrasound-activation has been achieved 50% of drug release in 2 h and 90% of drug release was achieved in 12 h, which displays more controlled release when compared to free RV solution. The *in vitro* cell compatibility analysis exhibited GCS-MONPs with RV has improved neuron cell survival rates when compared to other samples due to its porous surface and suitable biopolymer proportions. The analysis of *ex vitro* and *in vivo* pain relief analysis demonstrated treated animal models have high compatibility with GCS-MONPs@RV, which was confirmed by histomorphology. This developed MONPs based formulations with ultrasound-irradiation gives a prospective technique to clinical agony the board through on-request and dependable help with discomfort.

## 1. Introduction

Different nanosized drug cargo scaffold for local anesthetics (LAs) have been contemplated, including polymers, hydrogel, liposomes, etc. (Pathak & Nagarsenker, 2009; Alexander et al., 2012; Wang et al., 2013, 2016). Liposomes are likely the most outstanding cargo framework that has been utilized for the commercially accessible LA (Brown et al., 2006; Puglia et al., 2011). LA is broadly used to accomplish better agony control and lessening morbidity after the operation (Pignatello et al., 2009), yet the brief span of these medications cannot avoid the utilization of narcotics. Also, the duration and intensity of this treatment cannot be modified by the patient's requirement. While the constant infusion of LA employing a catheter can give long haul analgesia, it cannot be utilized in patients experiencing anti-coagulant treatment (Grant et al., 2001). Consequently, there is an acute requirement for developing another strategy for post-operative LA.

Contemporary ultrasound-activated medication conveyance system is for the most part centered on natural vehicles, including hybrids, micelles, and liposomes (de Araujo et al., 2013; Wakaskar, 2018), among which, liposomes have been demonstrated to be powerful in pain the management (Weiniger et al., 2010). Regrettably, natural particles like liposomes caught by the short half-life and low stability once infused are the inability to accomplish a long-haul pain-relieving impact. Recent years, mesoporous organo-silica nanoparticles (MONPs) have great attentions by nanotechnology and materials researchers for the numerous applications including drug delivery, purification, and catalysis, due to its promising abilities of tunable size, structural morphology, large pore volumes, surface-modifications, excellent cytocompatibility, tailorable degradation property, and particularly pure inorganic framework, which has been greatly helpful for the controlled release mechanism in drug delivery applications (Souza et al., 2014; Da'na, 2017). Importantly, different types of MONPs are involved in various drug delivery systems because they could be efficiently working as targeting delivery materials with mass diffusion, great transportation, and sustained manner. In addition, MONPs drug delivery system can be tailored through surface modifications, which also can be influenced by photo-irritation, pH, and redox reactions (Mbaraka et al., 2006; Terechova et al., 2014). In such manner, in this present work, MONPs, which have been featured to be steady and cytocompatibility both *in vivo* and *ex vitro* to research their ultrasound-activated supported discharging performance (Chakravarty et al., 2015).

Ropivacaine (RV) is a well-investigated long-acting and safest amide-based LA drug molecules, which has been applied alone and also combined with other drug materials (e.g. lidocaine, bupivacaine, etc.) for the active treatment of post-operative pain management after various surgeries (Grant & Bansinath, 2001; Zuo et al., 2004). The previous studies reported that, US Food and Drug Administration (FDA) has been approved RV hydrochloride injection from 1996, revealed that RV is highly suitable LA drug for surgery and severe pain management therapies. The usage of RV anesthesia has several clinical advantagous effects including long duration, reduced toxicity of central nervous and cardiovascular systems and greater sensory-motor differential blockade (Franz-Montan et al., 2007). RV, a clinically endorsed pain relieving with lower cardiovascular poisonous quality and restricted consequences for motor work at small concentrations, was effectively epitomized into MONPs by exploiting the porous conduit and porous inside morphology of MONPs (Shi et al., 2013).

Glycosylated chitosan (GCS) is a form of linear polysaccharide chitosan molecule which has been composed from the chitosan amine groups of d-glucosamine and N-acetyl-d-glucosamine by the β (1-4) linkage. These types of modifications in the chitosan molecular structure have been greatly influencing to improving aqueous solubility for the numerous biomedical applications such as photothermal cancer therapy, drug delivery, etc. (Xu et al., 1999; Zhou et al., 2011). Many recent research reports have elaborated that GCS is an effective immunoadjuvant agent that can be significantly applicable for targeted therapies for cancer treatments. The combination of photothermal and photodynamic techniques in cancer treatment would be gratefully efficient with GCS, which established that GCS would persuade immunological sensitivity for prevent tumor cells, which called laser immunotherapy treatment. In addition, that, GCS molecular structure have developed with some nanomaterials (e.g. carbon nanotube) for the effective photothermal therapy in immune-stimulated cancer therapies with excellent bio-degradability, low-toxicity with human cells (Chen et al., 2005).

The developed completely cytocompatibility component glycated chitosan encapsulated organo-silica nanoparticles loaded with RV (GCS-MONPs@RV) has been demonstrated to be exceptionally biocompatible, which has been methodically shown and assessed by both viability and rat model. It is outstanding that the microenvironment around harmed tissue is somewhat acidic. Also, upon ultrasound-activating, the raised and supported discharging of the drug was accomplished under ultrasound esteem *in vitro*. After the administration of MONPs@RV and GCS-MONPs@RV around the sciatic nerve, critical pain-relieving impact was distinguished in animal activity tests and histomorphological, examine, which likewise effectively mirrored the ultrasound activated enduring discharging of analgesic in the animal model.

## 2. Experimental

### 2.1. Fabrication of nano-biocomposite carrier

In a characteristic reaction, 1000 mg of cetyltrimethylammonium bromide was broken down in double distilled water under sonication and the solution temperature was acclimated to 100 °C. At that point, 2 M of NaOH solution was added to the cetyltrimethylammonium bromide arrangement. After consistent mixing, 5 mL of tetraethyl orthosilicate was included drop into the cetyltrimethylammonium bromide arrangement, and the reaction proceeded for another half a day. At that point, the strong rough item was acquired after the reaction blend was kept for in any event one day under calm conditions. This solid unrefined item was along these lines centrifuged, washed with distilled water and alcohol a few times and dried in a vacuum at 100 °C medium-term to get the free MONPs. 0.5 g of MONPs was centrifuged in the wake of refluxing in an alcohol hydrochloric acid arrangement at 50 °C for half a day to evacuate cetyltrimethylammonium bromide and was washed with DD and alcohol a few times and dried in hot air oven 80 °C for 24 h (Giraldo et al., 2007; Ma et al., 2013).

Functionalized MONPs was set up after the minor change of pervious works. Quickly, the readied MONPs were dried at 100 °C for 60 min. At that point, 1000 g of MONPs was blended into (3-aminopropyl) triethoxysilane. The subsequent specimens were confined by centrifugation, over and over washed, and dried in a hot air oven at 80 °C for 6 h. 0.05 g of GCS was disintegrated in the phosphate-buffered saline (PBS) support arrangement. At that point, 0.02 g functionalized MONPs was suspended into the GCS arrangement and blended at 50 °C for half a day. The suspension was in this way rapid centrifuged and dried at 80 °C medium-term. The acquired item was meant as GCS-MONPs (Chen et al., 2014).

### 2.2. Characterizations

SEM images were acquired utilizing a SU8020 and the specimens were not electrically conductive and required Au sputtering. XRD estimations were completed on an X-beam Diffractometer utilizing CuKa radiation (Geng et al., 2016). The FTIR spectra were gotten on a spectrophotometer in the scope of 400–4000 (She et al., 2015). N_2_ absorptions were estimated on a Micromeritics surface and porosity analyzer at 77 K (Zaleski et al., 2016). The surface territory, pore volume, and pore size were determined to utilize the BET model and BJH technique.

### 2.3. *In vitro* drug release analysis

To examine RV drug release kinetic analysis, prepared composited samples with RV (0.75%) were wrapped into dialysis bags (molecular exclusion cellulose membrane; 12 K–14 kDa), and afterward, the sample containing dialysis bags were placed in 200 mL glass beakers containing biological PBS (pH 7.4) medium at 37 °C and stirred at 100 rpm. During experiment running, aliquot part of release medium (1.5 mL) was reserved from the total release medium at preferred time intervals and withdrawn medium was replaced by fresh PBS to maintain sink condition. For the ultrasound-activating investigation, ultrasound with the individual power thickness of 1 and 2 W cm^−2^, and no activating as the standard was connected to the beakers by ultrasound coupling specialist secured test as recently set at the foreordained time. Finally, reverse phase HPLC was used to quantify of released drug molecules from the prepared nano-formulations. The discharge solution was monitored by UV absorption (240 nm) spectroscopic technique investigation to decide the measure of drug discharge (Foley et al., 2013; Zhang et al., 2017).

### 2.4. *In vitro* bio-compatibility assay

After one day of fibroblast (3T3) cells viability, well plates containing diverse biocomposite were put into various wells. The absorbance worth was perused at 450 nm by a UV absorbance microplate. The cell feasibility in the wells without biocomposite was set as a hundred percent and used to decide the relating cell suitability in the bio-composite treated cell.

### 2.5. *In vivo* animal studies

The *in vivo* animal model experiments were performed under the standard animal protocols approved by Animal care and Use Committee of Jinan Central Hospital, Shandong University, PR China and also followed the analgesia study protocols approved by international association of pain study. For the present investigation, male Sprague-Dawley rats were used with the weigh range between 300 and 350 g, and kept in cages with free food and water. The animals were anesthetized with 1, 2, 2, 2-tetrafluoroethyl difluoromethyl ether for receiving infusions. The strategy for exposing the sciatic nerve in the animal model of perpetual chronic constriction injury was performed in the present investigation. The left rear leg was shaved and sterilized. At that point, a cut was made along the lower edge of the femur to uncover the muscles underneath. Obtuse canalization was executed to uncover the sciatic nerve, which was delicately withdrawn. The animal models were administered through injectable with prepared RV loaded nanoformulations (0.5 mL). After suture, the entry point was secured with anti-microbial balm. For ultrasonic setting off, the animal was set in an independent cylinder with the rear leg uncovered, and after that, an ultrasonic test covered with gel was connected straightforwardly to the infused territory utilizing the settings as recently depicted (1 W cm^−2^, 1 MHz, 1 min).

### 2.6. Behavioral test

Warm hyperalgesia was estimated utilizing the Hargreaves mechanical tests were done 0–200 min. The animals were set in a chamber with a glass warming plate. After convenience for half an hour, the experiments were performed on the plantar outside by focusing a focused, glowing warmth light source on the highest point of the glass to make an extreme dot on the paw. PWL appeared on the showcase until the withdrawal of rear paw. The contralateral rear paw was tried as recently portrayed. Every rear paw was estimated for multiple times, and the normal was determined (Galley et al., 2017).

### 2.7. *In vivo* biocompatibility assay

The animal that got an infusion of prepared samples with or without seven cycles of ultrasonic irradiation 7 and 14 days after infusion. The tissue around the sciatic nerve was reaped and put away in 4% paraformaldehyde medium-term. At that point, the specimens were dried out utilizing reviewed ethanol and implanted with paraffin. Following cutting into segments, the cuts were de-waxed and re-colored with H&E. Optical microscopy was utilized to describe the *in vivo*-compatibility at the infusion location.

## 3. Results and discussion

### 3.1. Physicochemical analysis

**Figure 1.**
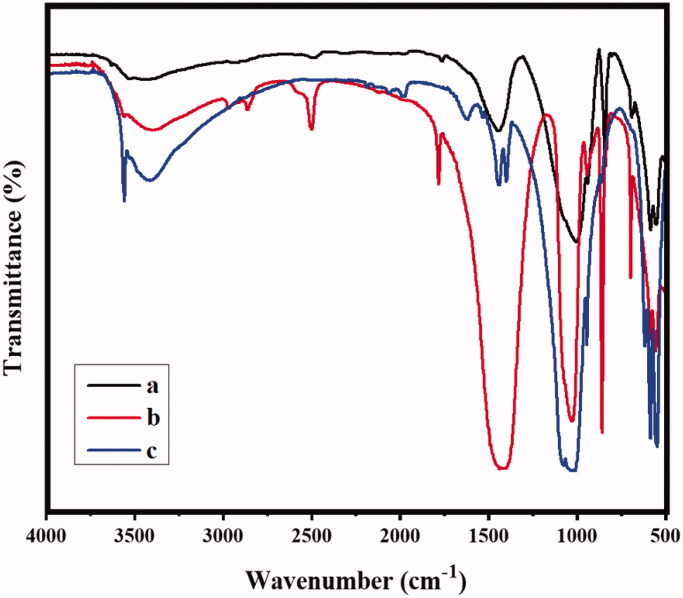
The analysis of molecular interaction and chemical structure by the method of FTIR spectral technique; (a) MONPs, (b) MONPs@RV, and (c) GCS-MONPs@RV samples.

**Figure 2.**
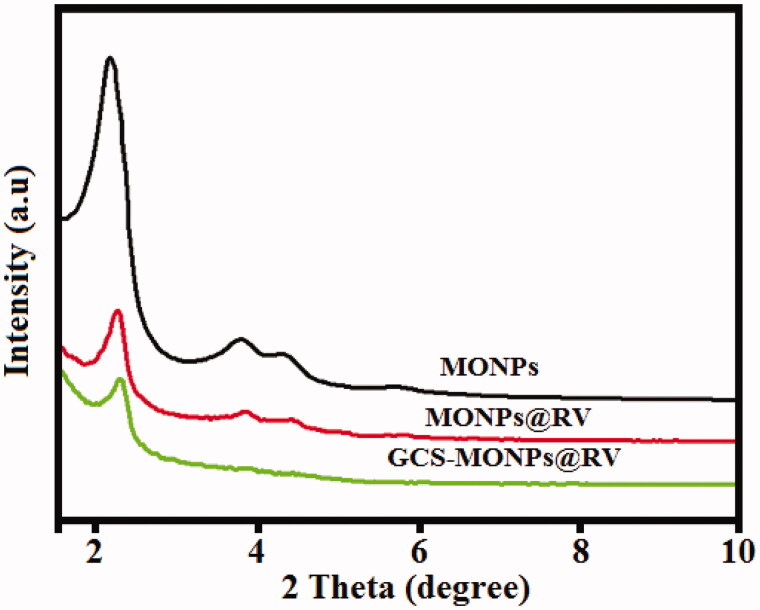
The analysis of phase purity and crystalline nature of prepared nanoformulations by the technique of XRD pattern analysis.

The FTIR spectra of MONPs, MONPs@RV, and GCS-MONPs@RV are shown in Figure 1. All the prepared specimens demonstrated bands at 1649, 810, and 496 cm^−1^ due to Si–O bending and stretching, respectively. Contrasted and chitosan (Murali et al., 2018), Figure 1(b,c) demonstrates the bands of amides I and II of GC exhibited at the peak ranges of 1626 and 1531 cm^−1^, indicating that bathochromic move, which revealed that MONPs was covered by the GCS (Yu et al., 2015). Additionally, an extra band displays in the MONPs@RV and GCS-MONPs@RV at 1580 cm^−1^ because of the amide bowing which is missing in GCS-MONPs. This results clearly demonstrated that successful functionalization of MONPs. Additionally, FTIR examination of GCS-MONPs and GCS-MONPs@RV established that the retentions of amide and methyl bonds were related to the atomic structure of the RV productively loaded into GCS-MONPs (Figure 2(c)).

### 3.2. XRD spectrum

Figure 2 shows the XRD diagrams of MONPs, MONPs@RV, and GCS-MONPs@RV. XRD of MONPs demonstrates an exceptionally extreme diffraction top at 2 thetas and three powerless planes at 2 theta values running from 3 to 6 that can be recorded as 210, 200, 110, and 100 which show hexagonal pore morphology (Liu et al., 2015; Wu et al., 2015). For MONPs@RV and GCS-MONPs@RV, three trademark XRD planes, 200, 110, and 100, are available which show that the arranged porous morphology of MONPs was held after alteration with GCS and RV groups. Though, the diffraction intensity diminished, affirming the effective change by GCS and RV groups (Zhu & Shi, 2007).

### 3.3. Morphology analysis

**Figure 3.**
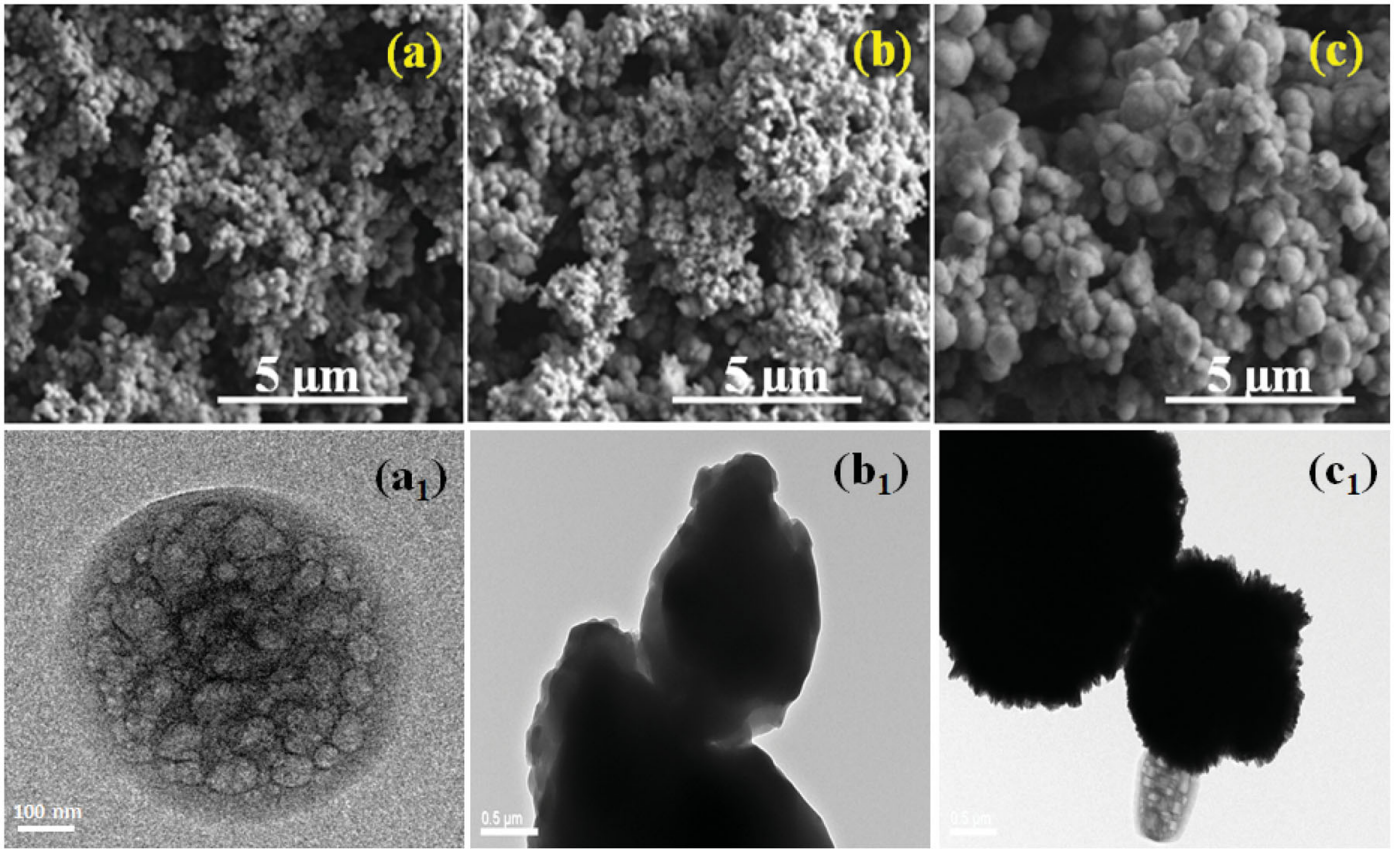
SEM and TEM images were exhibited as MONPs (a, a_1_), MONPs@RV (b, b_1_), and GCS-MONPs@RV (c, c_1_) nanoparticles, respectively.

Representative SEM and TEM pictures of prepared samples are appeared in Figure 3, demonstrating that the permeable surface and texture of the nanoparticles were not influenced by the encapsulation system. Based on our investigation on MONPs as medication cargo, we would thus be able to surmise that similar holds for the other prepared samples examined in this work. The pictures demonstrate that the particles semi-round structure and permeable structure are saved after encapsulation, without obvious surface alteration.

### 3.4. TGA and N_2_ adsorption investigation

**Figure 4.**
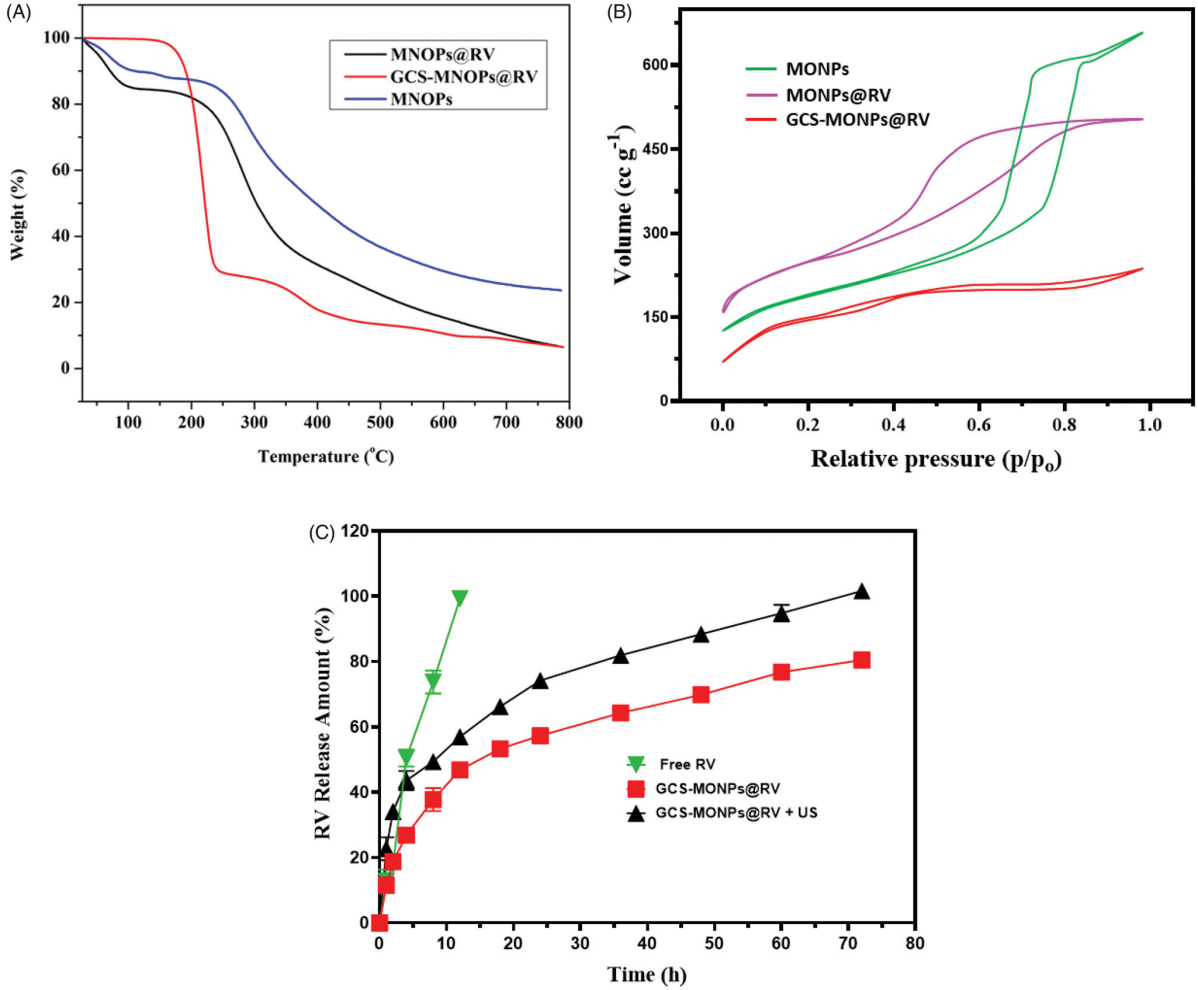
(A) TGA curve, (B) N_2_ sorption isotherms, and (C) ultrasonic-activated on-demand RV drug-releasing pattern from prepared nano-formulation of MONPs, GCS-MONPs, and GCS-MONPs + US (ultrasound activation) samples.

Further affirmation of the drug loading and functionalized of MONPs was shown in the thermogravimetric investigation. Figure 4(A) demonstrates the rate of mass loss profiles as a component of temperature for MONPs@RV and GCS-MONPs@RV. It tends to be seen that in the wake of warming the specimens up to 800 °C, MONPs, MONPs@RV, and GCS-MONPs@RV demonstrate a mass loss of ∼7.4, 13.8, and 34.9% individually. From these outcomes, the rate uniting of RV onto MONPs was determined to be ∼13.5%.

N2 adsorption–desorption isotherms of MONPs, MONPs@RV, and GCS-MONPs@RV demonstrated type 4 isotherms, which showed the porous nature of MONPs as appeared in Figure 4(B). BJH strategy was utilized for the pore size examination (Kachbouri et al., 2018). MONPs demonstrated a pore size of ∼4 nm, which diminished to 2.5 nm on functionalization. This little diminishing in the pore size could be because of the nearness of certain functionalization bunches inside the pores. The pore size was additionally diminished to 2.0 nm on account of MSN-GMC because of fruitful consolidation of the drug inside the pores of MONPs. The drug joining additionally brought about a lessening of surface territory and the pore volume of MONPs as appeared Figure 4.

### 3.5. Drug release study

To investigate the *in vitro* drug release kinetics of the prepared MONPs@RV and GCS-MONPs@RV nanostructures under ultrasound irradiation as shown in Figure 4(C). The drug releasing rate of the RV loaded nanostructures was evaluated and demonstrated with ultrasound-irradiation activated power forces of 5 W cm^−2^ and samples without ultrasound-irradiation has been considered as standard (Figure 4(C)). The analysis of drug releasing study has been displayed that prepared nanoformulation given greater and sustained release profile under 5 W cm^−2^ ultrasound-activated power when compared to the absence of ultrasound irradiation. These evaluation results demonstrated that prepared MONPs nanostructures have been greatly responsive to ultrasound irradiation and also influenced by porous surface modifications. Prominently, the drug release profile of GCS-MONPs@RV under ultrasound-irradiation process clearly revealed that improved sustained release of drug materials and importance of ultrasound activation in the present study, because mechanical and cavitation’s impact of ultrasound-activation influencing between the connections of drug molecules and MONPs and lead to the convenient breaking for the delivery (Rahman et al., 2017). In addition, the drug release profile of prepared nanostructure (GCS-MONPs@RV) with ultrasound-activation exhibited 50% of drug release in 2 h and 90% of drug release was achieved in 12 h, which is established faster than MONPs@RV sample and more controlled release when compared to free RV solution. This *in vitro* drug release kinetics indicated that the prepared nano-formulations would probably contribute sustained and increased drug distributions in post-surgery LA therapies with actions of targeting site and long-lasting effect.

### 3.6. Biological study

#### 3.6.1. *In vitro* biocompatibility

**Figure 5.**
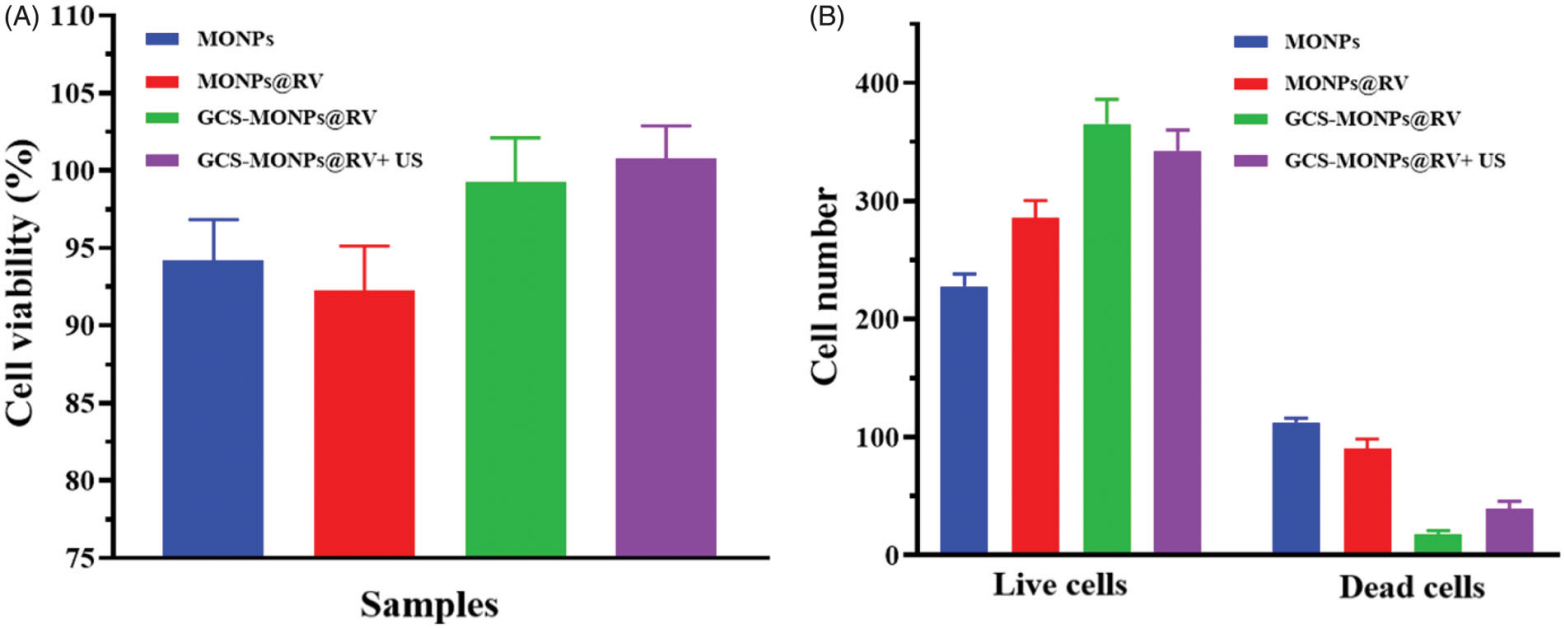
(A) *In vitro* quantitative results of cell viability and (B) quantitative cell number of live and dead cells calculated from cellular fluorescence images.

In this examination, we have used fibroblast (3T3) and human keratinocyte (HACAT) cell lines to assess the cytocompatibility of the prepared composite. The cell biocompatibility of both human cells (3T3 and HACAT) in composites with the MONPs, MONPs@RV, and GCS-MONPs@RV was estimated by MTT assay and the estimation of optical thickness are relative to the number of live cells. As appeared in Figure 5(A), the general pattern demonstrated that cell survival rates on MONPs composite were lower than those of GCS-MONPs@RV which had higher porosity and biopolymer proportion. Three kinds of prepare materials all demonstrated a slowly upward pattern of cell number over culture time amid 1–5 days, showing that the composite could bolster cells survival and multiply. Among the porous composite gatherings, the best number of joined cells was found in the GCS-MONPs@RV. Besides, there was no critical distinctive in cell number between MONPs@RV and MONPs frameworks.

**Figure 6.**
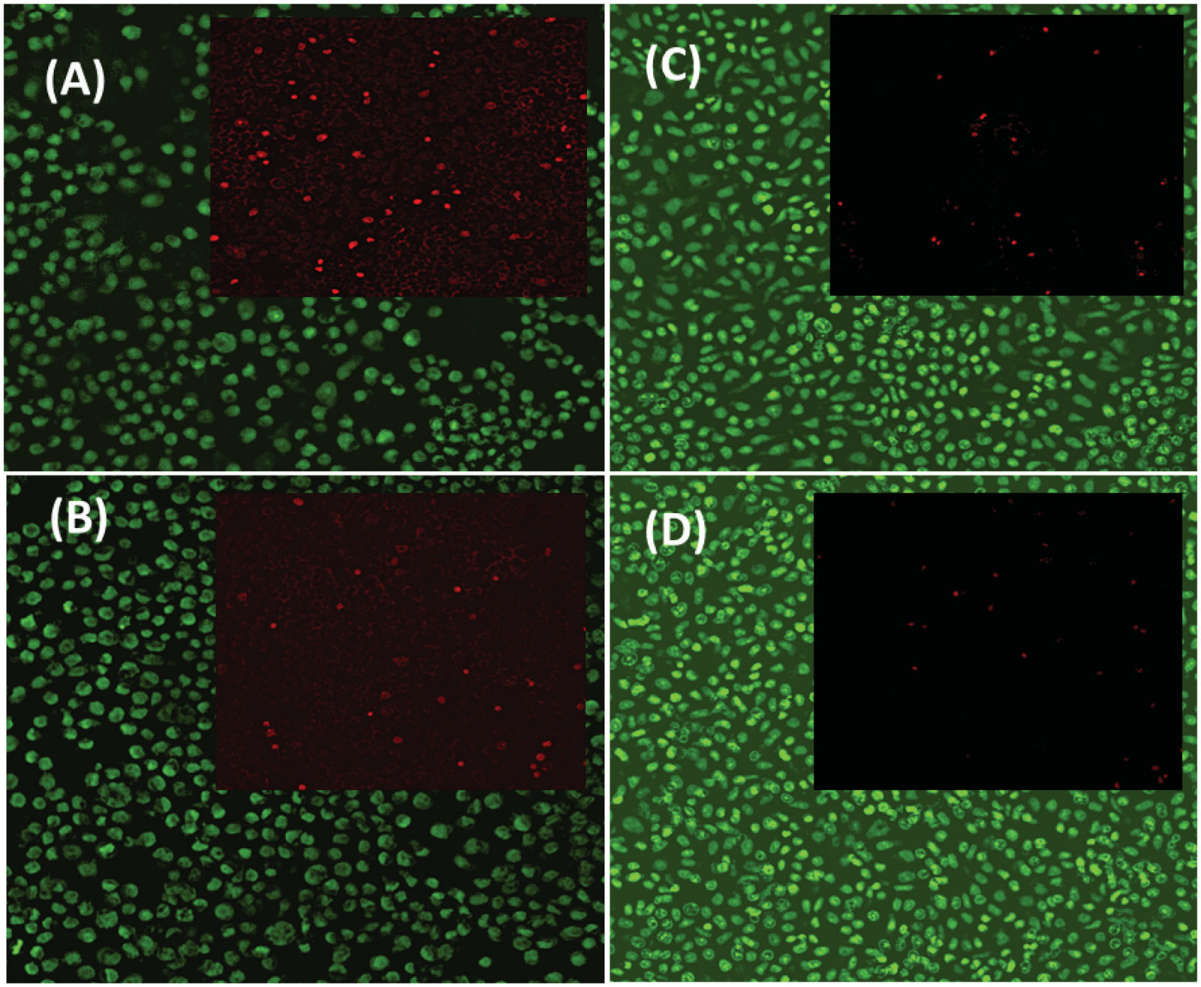
*In vitro* immunofluorescence images of live (green) and dead (red) viability analysis of 3T3 fibroblast cells on prepared nanostructured samples of (A) MONPs, (B) MONPs@RV, (C) GCS-MONPs@RV, and GCS-MONPs@RV + US.

As noted in Figure 6, the live cells on prepared samples were stained with green fluorescence through the dead cells were recolored red. Contrasted with MONPs, MONPs@RV and GCS-MONPs@RV demonstrated progressively alive cells, as found in Figure 5(B). For synergistically GCS-MONPs@RV, the thickest dissemination profile of neuron cells was watched (Figure 5). Quantitative investigation of live/dead cell numbers on these three type of materials indicated the most astounding number of neuron cells on the GCS-MONPs@RV with the least number of cells on nonpartisan MONPs. As can be seen from Figure 6, all the prepared samples showed essentially good viability to neuron cells.

#### 3.6.2. *In vivo* ultrasonic-active

**Figure 7.**
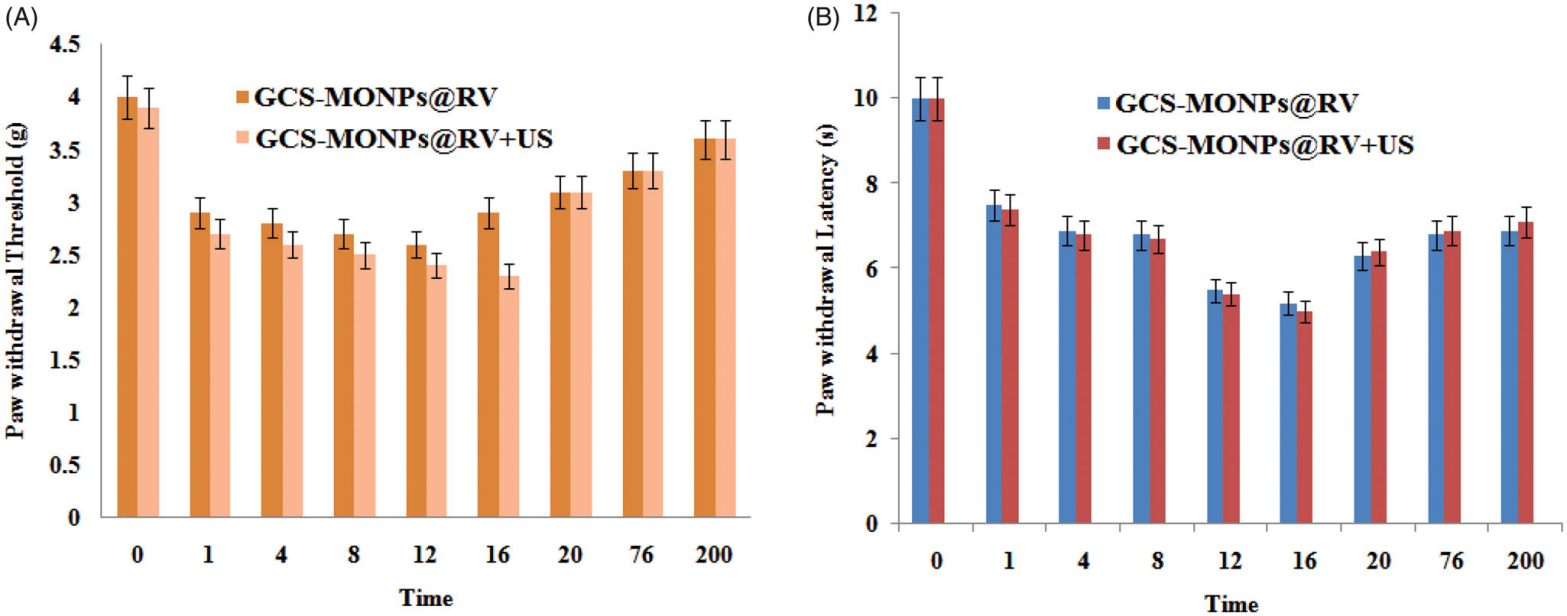
Representative time courses of pain assessments: (A) threshold and (B) latency profile after treatment.

As we expected, no noteworthy impact on pain relief has distinguished both gatherings (Figure 7(A,B)), further exhibiting that scratch point torment was obstructed by the planned ultrasonic responsive discharged drug from composite. The impression in the contra-lateral paw did not change in the drug and biocomposite bunches in each activating cycle, recommending an absence of a foundational sedate appropriation. The mechanical threshold and warm inertness slowly came back to the pattern in all gatherings following multi-week, showing the normal activities of the *in vivo* model.

#### 3.6.3. *In vivo* compatibility assay

**Figure 8.**
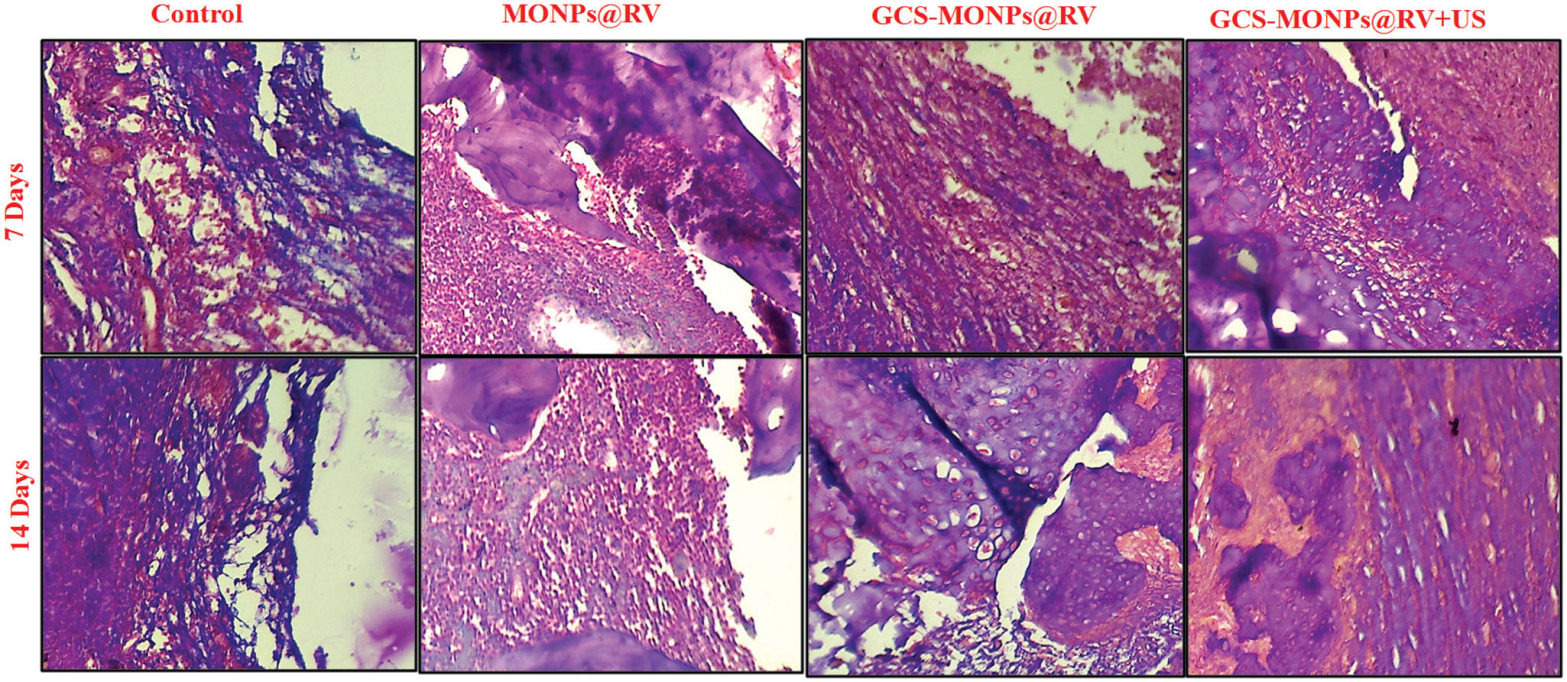
H&E-stained sections of animal sciatic nerve different weeks after the treatment with prepared samples with or without of ultrasonic activation.

To reveal the potential *in vivo* biocompatibility of prepared samples pain treatment, rats that got infusions of GCS-MONPs@RV with or without US irradiation were euthanized seven days and 14 days after infusion. Histomorphology of H&E recolored tissue areas showed no conspicuous harmful reaction happened amid treatment (Figure 8); however, there happened incendiary cell penetration, which is usually seen with infused samples (Gupta et al., 2017). There was no distinction in bio histocompatibility between GCS-MONPs@RV with or without US irradiation, and the kind irritation was diminished 14 days after infusion. Histocompatibility was high in every single test gathering. Hence, these outcomes showed the high histocompatibility of GCS-MONPs@RV as nano-scaffold both *ex vitro* and *in vivo* for pain relief.

## 4. Conclusions

In conclusion, we have prepared, an infusible and porous system that gives delayed term of analgesia because of its highlights of good reaction to ultrasound and continued medication discharging from the porous-carriers, which could accomplish the defeating of the inadequacies of customary pain therapy and improve the remedial effectiveness of local analgesics. The presence of well-characterized porous morphology and huge hollow hole of MONPs empowers high medication stacking limit, making them profoundly potential for accomplishing proficient treatment of torment. The discharging amount of stacked medication is upgraded via ultrasound, which would be helpful for during patient-controlled absence of pain and accomplishing better relief from discomfort in incision areas. The viability and live/dead cell assay measure have exhibited the high restorative biocompatibility of this on-request porous system for enduring and acceptable help with discomfort *in vivo*. Moreover, the high biocompatibility and non-neurotoxicity of these nano-scaffolds were additionally illustrated, ensuring the prospective for further clinical interpretation. This work not just essentially expands the utilization of MONPs as porous-carriers in pain management, yet also gives an effective helpful methodology on pain control by ultrasound activating with dependable and on-request features.
